# Prediction of Major Histocompatibility Complex Binding with Bilateral and Variable Long Short Term Memory Networks

**DOI:** 10.3390/biology11060848

**Published:** 2022-06-01

**Authors:** Limin Jiang, Jijun Tang, Fei Guo, Yan Guo

**Affiliations:** 1 Comprehensive Cancer Center, Department of Internal Medicine, University of New Mexico, Albuquerque, NM 87131, USA; jianglm@tju.edu.cn; 2 Shenzhen Institute of Advanced Technology, Chinese Academy of Sciences, Shenzhen 518055, China; jtang@cse.sc.edu; 3 School of Computer Science and Technology, College of Intelligence and Computing, Tianjin University, Tianjin 300350, China

**Keywords:** major histocompatibility complex, bidirectional long short-term memory neural network, deep learning

## Abstract

**Simple Summary:**

Major histocompatibility complex molecules are of significant biological and clinical importance due to their utility in immunotherapy. The prediction of potential MHC binding peptides can estimate a T-cell immune response. The variable length of existing MHC binding peptides creates difficulty for MHC binding prediction algorithms. Thus, we utilized a bilateral and variable long-short term memory neural network to address this specific problem and developed a novel MHC binding prediction tool.

**Abstract:**

As an important part of immune surveillance, major histocompatibility complex (MHC) is a set of proteins that recognize foreign molecules. Computational prediction methods for MHC binding peptides have been developed. However, existing methods share the limitation of fixed peptide sequence length, which necessitates the training of models by peptide length or prediction with a length reduction technique. Using a bidirectional long short-term memory neural network, we constructed BVMHC, an MHC class I and II binding prediction tool that is independent of peptide length. The performance of BVMHC was compared to seven MHC class I prediction tools and three MHC class II prediction tools using eight performance criteria independently. BVMHC attained the best performance in three of the eight criteria for MHC class I, and the best performance in four of the eight criteria for MHC class II, including accuracy and AUC. Furthermore, models for non-human species were also trained using the same strategy and made available for applications in mice, chimpanzees, macaques, and rats. BVMHC is composed of a series of peptide length independent MHC class I and II binding predictors. Models from this study have been implemented in an online web portal for easy access and use.

## 1. Introduction

Major Histocompatibility Complex (MHC) genes code for proteins that recognize foreign molecules and play an important part in immune surveillance. Due to variation in molecular structure, function, and distribution, MHC molecules are divided into three subsets: MHC class I, II, and III. A MHC class I molecule may constitute the MHC heavy chain (alpha chain), which encompasses three alpha domains (alpha1, alpha2, and alpha3) [1]. Alpha1 and alpha2 form the recognition region, with an interval deep groove capturing the peptide antigen [2]. Alpha3 is adjacent to the transmembrane domain in the heavy chain and it interacts with antigen transporters to load and express antigens. A specific type of MHC class I molecules are encoded by the β2-microglobulin gene, and in MHC they constitute the MHC light chain (beta chain). MHC class I molecules are located at the surface of cells to present antigens, which trigger immune responses by attracting cytotoxic lymphocytes immune cells (TC cells) including CD8+, the cytotoxic T cells which express CD8+ receptors. These receptors recognize related MHC complexes at the cell surface: when an antigen peptide of foreign origin is bound, CD8+ immune cells are activated to trigger programmed apoptosis [1]. An MHC class II molecule encodes two membrane-spanning chains that are of similar size. While MHC I molecules are located on the surface of nearly all nucleated cells, MHC II glycoproteins are expressed on the surface of specialized immune cells (such as B cells, macrophages, and dendritic cells), where they present processed antigenic peptides to TH cells. MHC class III genes encode various secreted proteins that have immune functions, including components of the complement system and molecules involved in inflammation [3].

Of the three MHC classes, class I has attracted great attention in medical research. For example, reduced abundance in MCH class I is associated with poor prognosis in Hodgkin lymphoma [4]. Another study [5] demonstrated that cancer cells escape T-cell responses via losing MHC class I molecules. MHC molecules are highly polymorphic proteins. As one MHC protein can have many variants, and such variants are commonly referred to as “MHC alleles” [6], MHC alleles are organized into multiple categories for each MHC class. For instance, MHC class I proteins in humans are encoded as human leukocyte antigen (HLA) groups A, B, C, etc. by the gene name, and each HLA group is composed of many alleles by the variants. From the view of molecular structure, MHC molecules have pockets, and the antigenic peptides have anchors of which some are determined residues, and anchors have special properties to lead peptides to enter the pockets [7]. An antigenic peptide’s MHC binding affinity can be measured experimentally by a variety of assays, including a competitive binding assay [8].

The accumulated experimentally verified MHC binding peptides have been curated into various databases during the last three decades. Around 13 MHC binding databases are currently available [9]. With more than 900,000 entries, the Epitope Database (IEDB) [10] contains the largest collection of MHC binding peptides, followed by MHCBN [11] curating 25,860 peptides. In addition to the experimental methods, a peptide’s binding potential with regard to a particular MHC molecule can be estimated through computational algorithms. Computational methods can systematically prioritize credible candidates for a more favorable study design, thus helping reduce both financial cost and human labor of the wet-lab assay-based validation experiments. The experimentally verified MHC binding peptide sequences offer an understructure for the development of computational approaches to predict the binding affinity between an MHC allele and a novel peptide. More than 30 MHC binding prediction tools were developed based on the accumulated MHC binding databases over the years. The majority of these tools [12,13,14,15,16,17,18,19,20,21,22,23,24] were developed for MHC class I and II binding prediction.

A common limitation of the existing MHC binding prediction tools is the necessity to align all peptides to one fixed length. Specifically, to meet the requirement, developers must either train different models to tackle peptides of different lengths, or they must arbitrarily adjust the original peptide. There are two sequence selection strategies in the model training/predicting process, one of which is to select peptides with a fixed length, such as selecting 9-mer peptides to train a model for class I [25,26,27]. The other is to adjust the peptides sequence to a specific length, such as adjusting the peptide length of class I to 9-mer/15 mer by inserting “X” symbols (elongating) or deleting amino acids (shortening) [28,29,30,31]. For the first strategy, there are two disadvantages: (1) It is tedious to train multiple models out of the initial single allele set; (2) When dividing the whole training set into multiple length-specific training sets, some models of certain lengths may have insufficient training data and therefore result in undertraining and suboptimal performance. For the second strategy, one obvious disadvantage is that inserting or deleting amino acids inevitably leads to a loss of information; specifically, the neighbor amino acids at a perturbed position will not be the same post the elongating/shortening operation. To overcome this constraint, we developed BVMHC, a novel MHC binding prediction tool based on Bidirectional Long Short-Term Memory (biLSTM) neural network [32,33], a type of recurrent neural network (RNN), which has the major novelty of offering variable length MHC binding prediction. BVMHC is designed to make predictions for both MHC class I and class II alleles in humans, and models for non-human species were also trained using the same strategy. The performance of BVMHC has been thoroughly compared with popular MHC class I/II binding prediction tools.

## 2. Materials and Methods

### 2.1. Training and Validation Datasets

To establish a sizeable training dataset, we obtained from the IEDB database 122,129 and 45,440 human binding peptide sequences for 48 MHC class I alleles and 27 MHC class II alleles, respectively. Additionally, 15,740 MHC class I peptide sequences of four non-human species (mouse, rat, macaque, and chimpanzee) and 1041 MHC class II peptide sequences of mouse were also extracted from the IEDB database. Each peptide was associated with a binding affinity measured as ${\mathrm{IC}}_{50}\;$in *nM*. A dichotomization of these binding affinity values was conducted as follows: peptides with ${\mathrm{IC}}_{50}\;\geq 500\;nM$ were considered as negative binding and peptides with ${\mathrm{IC}}_{50}<500\;nM$ were considered as positive binding. All binding affinity values (*aff*) were standardized to the interval [0,1] through a function, i.e., 1−log (aff)/log(50,000). The initial sequences underwent the following three aspects of filtration: (1) For sequences that are repeated and have the same IC50 value, we kept only one instance of the sequences and removed all duplicate instances. (2) For sequences that are repeated and have different IC50 values, we deleted all items. (3) For sequences that are repeated and have different allele information, we kept all items because we would train different predictors for different allele sequences. Five-fold cross-validation procedures were used on the training datasets to train models. An independent validation dataset consisting of 320 class I and 131 class II human peptide sequences was constructed from the databases MHCBN [34] and SYFPEITHI [35], where we made sure that items co-existing in the IEDB were removed.

### 2.2. Feature Representation at Evolutionary Level

**Figure 1:**
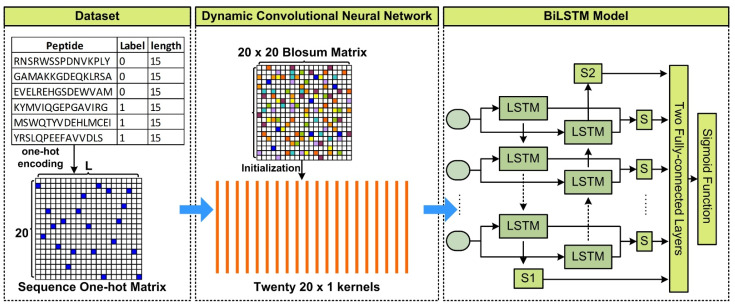
Overview of BVMHC. One-hot encoding was used to convert a peptide sequence to a matrix. BLOSUM was applied to initialize kernels in the convolutional neural network that was used to extract the peptide sequence feature at the evolutionary level. The biLSTMmodel was then applied to process the merged matrix at the sequential level.

The BVMHC model involves two major components: feature representation and the computation model (Figure 1). For a numerical representation of training/testing data, each peptide sequence was first encoded as a 20×L matrix through one-hot encoding [36], where *L* is the length of the peptide. The dynamic convolutional neural network with twenty 1×20 convolution kernels was used to process one-hot coding matrices. BLOSUM is a 20×20 matrix that represents evolutionary conservation information between amino acids [37], and we used it to initialize the twenty convolution kernels. The overall method can be represented with Equation (1), where *X* denotes the One-hot encoding matrix, *i* the index of amino acid in peptide, *k* the index of kernel, *M* = 1 the window size, and *n* = 20 the number of kernels. Of note, the two indices, *i* and *j*, start from an initial value of 0.

(1) $$\mathrm{Evo}{\left(X\right)}_{i,k}=\sum_{m=0}^{M-1}\sum_{m=0}^{m=0}W_{m,n}^{k}X_{i+m,n}\;$$

As the kernels were updated in the training process, an updated presentment matrix in the evolutionary level was obtained and was input into a biLSTM model. After training, a novel BLOSUM matrix can be obtained by using the twenty trained convolution kernels.

### 2.3. Feature Representation at Sequential Level

The advantage of biLSTM (Figure 1) is the ability to handle peptides with variable lengths. Long short-term memory (LSTM) [33] is a type of recurrent neural network and all connections between units in LSTM form a directed cycle. This cycle is conducive to modeling dynamic temporal or spatial behavior. LSTM block is dynamically changed with the sequence length. An LSTM unit includes input, forget, and output gates. The calculation process is defined as Equations (2)–(6), where, $x_{t}$ denotes the input vector, $f_{t}$ the forget gate’s activation vector, $o_{t}$ the output gate’s activation vector, $h_{t}$ a 128-dimenstion hidden state vector, and $C_{t}$ the cell state vector. In these equations, the common notations *W* and *U* refer to parameter matrices and *b* designates a bias vector.

(2) $$f_{t}=\sigma \left(W_{f}x_{t}+U_{f}h_{t-1}+b_{f}\right)$$

(3) $$i_{t}=\sigma \left(W_{i}x_{t}+U_{i}h_{t-1}+b_{i}\right)$$

(4) $$o_{t}=\sigma \left(W_{o}x_{t}+U_{o}h_{t-1}+b_{o}\right)$$

(5) $$C_{t}=i_{t}{}^{\circ}\tanh\left(W_{c}x_{t}+U_{c}h_{t-1}+b_{c}\right)+f_{t}{}^{\circ}C_{t-1}$$

(6) $$h_{t}=o_{t}{}^{\circ}\tanh\left(C_{t}\right)$$

In our biLSTM model, one set of LSTMs merged the feature matrix from left to right, and another set of LSTMs merged the feature matrix from right to left. A dropout layer was applied to avoid over-fitting. A vector with 128 dimensions from biLSTM was obtained first. Afterward, a regression output value was obtained from two fully-connected layers and converted into a probability through the sigmoid function. In the process of training, we chose binary cross-entropy as the loss function and set the learning rate at 0.0001, and the dropout rate at 0.8.

### 2.4. Evaluation Criteria

Eight evaluation criteria, including Accuracy, Sensitivity, Specificity, F1, Matthew’s correlation coefficient (MCC), Precision, Area Under the receiver-operating-characteristic Curve (AUC), and Area Under the Precision-Recall curve (AUPR), were used to evaluate the performance of the models. The calculation of the first six criteria is illustrated in Equations (7)–(12), where *TP* represents the number of true positive MHC binders, false negative represents the number of true negative MHC binders, *FP* represents the number of false positive binders, and false negative represents the number of false negative MHC binders.

(7) $$\mathrm{Accuracy}=\frac{TP+TN}{TP+TN+FP+FN}$$

(8) $$\mathrm{Sensitivity}=\frac{TP}{TP+FN}$$

(9) $$\mathrm{Specificity}\;=\frac{TN}{TN+FP}$$

(10) $$F1=\frac{2\times \left(\mathrm{Precision}\times \mathrm{Sensitivity}\right)}{\left(\mathrm{Precision}+\mathrm{Sensitivity}\right)}$$

(11) $$\mathrm{MCC}=\frac{TP\times TN-FP\times FN}{\sqrt{\left(TP+FP\right)\times \left(TN+FN\right)\times \left(TP+FN\right)\times \left(TN+FP\right)}}$$

(12) $$\mathrm{Precision}=\frac{TP}{TP+FP}$$

## 3. Results

### 3.1. Human Dataset Description

**Figure 2:**
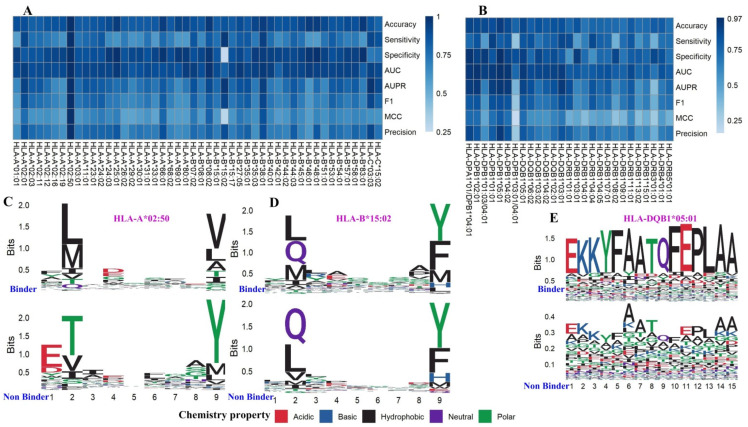
BVMHC performance on human datasets and binding motifs of a few extremity models. (**A**,**B**) The performance of BVMHC on the training dataset for predicting human MHC Class I (**A**) and II binders (**B**) in five-fold cross-validation. (**C**) The motifs of binders and non-binders for MHC Class I allele HLA-A*02:50. (**D**) The motifs of binders and non-binders for MHC Class I allele HLA-B*15:02. (**E**) The motifs of binders and non-binders for MHC Class II allele HLA-DQB1*05:01.

The numbers of binding (positive examples) and non-binding (negative examples) peptides for MHC class I and II alleles making up the training and independent validation datasets are available in Supplementary Table S1. Overall, the human training dataset consisted of 75 alleles and entailed multiple (n) distinct peptide sequence lengths. For each of the 75 alleles, traditional approaches would have trained n length-dependent models to tackle different peptide lengths, or trained one fixed-length model which would necessitate a pre-procedure of length adjustment. Using the length-independent approach biLSTM, we trained 75 length-independent models and validated them with five-fold cross-validation. All 48 models for MHC class I binding and 12 of 27 models for MHC class II binding achieved over 0.8 accuracy and AUC values (Figure 2A,B). Overall, there exists a considerable difference in the performance levels between MHC Class I and Class II models, with the latter exceeding the former. Performances of MHC Class I models are generally acceptable except for a few outliers, such as HLA-B*15:02.

We identified a few models of extremity performances and went on to characterize the sequence motifs. Specifically, the performance values of HLA-DQB1*05:01 in Figure 2B and HLA-A*02:50 in Figure 2A are nearly one. By contrast, the MCC and Specificity associated with HLA-B*15:02 in Figure 2A are merely 0.25. We analyzed the difference between motifs of Binders and Non-Binders for HLA-A*02:50, HLA-B*15:02, and HLA-DQB1*05:01 (Figure 2C–E), respectively. Figure 2C,E describe the motifs for the well-performing models HLA-A*02:50 and HLA-DQB1*05:01, and we can see that the amino acid motifs are distinct between Binders and Non-Binders. Figure 2D describes the bad-performing model HLA-B*15:02, which shows non-differential motifs between Binders and Non-Binders. Therefore, the unsatisfactory prediction performance might be due to the weak distinction in motif patterns between positive and negative examples, which may hint at the contamination of binders by many false positives (non-binders). The good performance of BVMHC is attributed to the exploitation of the positional conservation and the preservation of intact peptide sequences. The detailed performance evaluation results by peptide length can be found in Table 1.

### 3.2. Independent Validation and Comparison with Other MHC Binding Predictors

**Figure 3:**
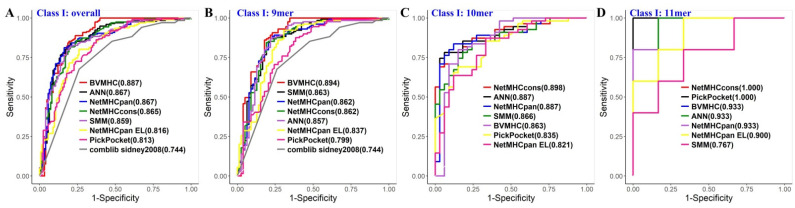
Receiver-Operating-Characteristic (ROC) curves of the eight tools for predicting MHC class I binders on the independent validation dataset. (**A**–**D**). BVMHC and seven existing prediction tools for overall (**A**), 9-mer (**B**), 10-mer (**C**), and 11-mer (**D**) MHC class I binders, respectively.

An independent dataset extracted from MHCBN and SYFPEITHIwas used for validation and comparison with other MHC binding predictors. Seven popular MHC class I binding predictors (comblib_sidney2008 [21], ANN [19], SMM [17], NetMHCcons [16], NetMHCpan [18], PickPocket [20] and NetMHCpan EL [24]) for class I and three well-accepted MHC class II binding predictors (NETMHCIIPan [23], NN-align [15] and SMM-align [22]) were selected for the comparison. A common limitation of these existing tools is that the established model is bounded by a fixed peptide sequence length, which means that investigators have to distort the sequence structure when they take special actions (insertion or deletion) to ensure that the peptide length meets the model requirement. Moreover, in the above section, we have demonstrated that a model’s prediction performance benefits from the positional conservation, the phenomenon of which is generally neglected in existing methods. The performance of BVMHC and the other MHC class I/II tools was measured with the eight aforementioned criteria (Table 2), and the complete results are displayed in Table S2. Models of HLA-DRB1*03:01 trained to predict MHC class II binding peptide achieved accuracy and AUC over 0.8 on the five-fold cross-validation; we downloaded the HLA-DRB1*03:01 peptide data from MHCBN. Of all eight evaluation indices, BVMHC achieved the best performance in three of the eight criteria for MHC class I prediction and the best performance in four criteria for MHC class II prediction. For example, BVMHC obtained the best overall AUC of 0.887 (Figure 3A), and the best average AUC for 9-mer models in MHC class I prediction (Figure 3B).

### 3.3. Performance of Non-Human Species

Using the same strategy as in humans, BVMHC models were also trained for MHC class I prediction for three mouse alleles, eight macaque alleles, five chimpanzee alleles, and one rat allele; in addition, two mouse MHC class II alleles were also covered. Results of five-fold cross-validation of these non-human MHC prediction models are available in Table 3. All 17 MHC class I models achieved greater than 0.8 accuracy and AUC. Both MHC class II models obtained greater than 0.80 accuracy. Due to the limitations of non-human data availability, independent validation was not performed.

### 3.4. Web Server Implementation

A web server for the BVMHC models was developed using the combination of R, PHP, and Python, which is freely accessible at http://www.innovebioinfo.com/Proteomics/MHC/home.php. The website can conduct predictions for MHC class I and II binding peptides of multiple species. For MHC class I prediction, BVMHC covers 48 human alleles, three mouse alleles, eight macaque alleles, five chimpanzee alleles, and one rat allele; for MHC class II prediction, BVMHC covers 12 human alleles and two mouse alleles.

## 4. Discussion

MHC binding prediction is a crucial step toward identifying potential novel therapeutic strategies. For example, MHC class I molecules were found to be tumor suppressor genes [38] and can served as targets for immunotherapy [39]. Similar to MHC class I, the class II antigens can also serve as targets in cancer immunotherapy [40]. The prediction of MHC binding peptides is biologically and clinically important because it predicts the binding affinity of a T-cell immune response. Factors such as the polymorphic nature of MHC molecules, the variable length of peptides, etc. make it difficult to accurately predict MHC binding. However, advances in machine learning, especially those based on neural networks, have propelled substantial advancement in MHC binding prediction research. In this study, we proposed an approach using the Bilateral and Variable Long-Short Term Memory Networks to tackle the variable length issue in MHC binding prediction. By thoroughly comparing to other fixed-length-constrained MHC binding prediction tools, we show that BVMHC has the advantage in several performance measurements. However, In this paper, we just use the peptide sequences information to construct predictors. Inspired by NetMHCpan [18] and NetMHCIIpan [23], in the future we will incorporate the MHC protein sequence information to augment the feature representation of binders. As AlphaFold [41] becomes the focus of research about protein structure, we look to discern the differences between different MHC allele proteins at the protein structure level, which may hold promises for an even improved prediction of MHC protein binders. Additionally, a BVMHC predictor can be used to quickly screen potential binders—an effective strategy is to dissect a complete protein sequence into equal-sized segments and run the predictor over these segments across the whole span of the protein sequence. Considering the computational time complexity, such screening workflows must be optimized to reduce the running time to the minimum.

## 5. Conclusions

BVMHC is an MHC binding prediction tool that supports five species (human, chimpanzee, macaque, mouse, and rat). Compared to existing MHC prediction tools, BVMHC can use peptides of variable lengths to train a predictor, which allows for the reservation of the innate primary structure of the sequence. The combination of analyses at the conservatory level and the sequential level is vital for the superior performance of the resultant BVMHC model. In independent validation and comparison, BVMHC showed the best overall performance compared to seven other popular MHC class I predictors and three well-accepted MHC class II predictors. BVMHC was developed into a web server and can be accessed freely online.

## Figures and Tables

**Table 1:** Five-fold cross-validation results stratified by peptide length.

	Length	Accuracy	AUC	F1	MCC	Specificity	Sensitivity	Precision	AUPR	Positive ^1^	Negative ^2^
Class I	8 mer	0.891	0.924	0.783	0.531	0.887	0.677	0.525	0.785	229	1879
	9 mer	0.883	0.915	0.745	0.650	0.902	0.735	0.760	0.800	23,000	72,963
	10 mer	0.813	0.850	0.693	0.527	0.842	0.690	0.661	0.725	7263	14,024
	11 mer	0.879	0.905	0.768	0.608	0.881	0.756	0.651	0.755	310	1604
	Others	0.986	1.000	0.992	0.564	0.750	1.000	0.985	1.000	54	803
Class II	13 mer	0.857	0.879	0.883	0.700	0.833	0.872	0.895	0.923	232	205
	14 mer	0.898	0.907	0.880	0.792	0.912	0.880	0.880	0.873	131	239
	15 mer	0.868	0.906	0.781	0.687	0.912	0.769	0.794	0.840	16,743	25,683
	16 mer	0.776	0.846	0.802	0.545	0.718	0.823	0.782	0.878	563	569
	17 mer	0.680	0.673	0.429	0.312	0.933	0.300	0.750	0.643	106	257
	18 mer	0.643	0.939	0.706	0.452	1.000	0.545	1.000	0.986	71	40
	19 mer	0.875	0.938	0.857	0.775	1.000	0.750	1.000	0.950	55	75
	20 mer	0.750	0.900	0.500	0.488	1.000	0.333	1.000	0.886	65	66
	Other	0.690	0.640	0.381	0.183	0.758	0.444	0.333	0.566	81	259

^1^ Number of positives; ^2^ Number of negatives

**Table 2:** The comparison results of the BVMHC model against seven other prediction tools on the independent validation dataset. The best performance value in each comparison track is highlighted in bold text.

	Methods	Accuracy	Sensitivity	Specificity	AUC	AUPR	F1	MCC	Precision	Positive ^1^	Negative ^2^
Class I	BVMHC	0.597	0.371	**0.959**	**0.887**	0.866	0.531	**0.374**	**0.936**	197	123
	NetMHCcons [16]	**0.600**	**0.386**	0.943	0.865	0.890	**0.543**	0.365	0.916	197	123
	SMM [17]	0.584	0.350	**0.959**	0.859	**0.891**	0.509	0.357	0.932	197	123
	NetMHCpan [18]	0.566	0.330	0.943	0.867	0.886	0.483	0.318	0.903	197	123
	ANN [19]	0.563	0.325	0.943	0.867	0.880	0.478	0.314	0.901	197	123
	PickPocket [20]	0.563	0.345	0.911	0.813	0.833	0.493	0.289	0.861	197	123
	NetMHCpan EL [24]	0.553	0.335	0.902	0.816	0.856	0.480	0.269	0.846	197	123
	comblib_sidney2008 [21]	NAN ^§^	NAN ^§^	NAN ^§^	0.744	NAN ^§^	NAN ^§^	NAN ^§^	NAN ^§^	68	46
Class II	BVMHC	**0.878**	**0.333**	0.965	0.718	0.417	**0.429**	**0.386**	0.600	18	113
	NN-align [15]	0.863	0.278	0.956	**0.866**	**0.484**	0.357	0.303	0.500	18	113
	NETMHCIIPan [23]	0.870	0.111	**0.991**	0.795	0.423	0.190	0.235	**0.667**	18	113
	SMM-align [22]	0.840	0.000	0.973	0.787	0.319	NA ^§^	−0.061	0.000	18	113

^1^ Number of positives ^2^ Number of negatives ^§^ NA: the sum of Sensitivity and Precision is zero, thus F1 is NA. ^§^ NAN: the evaluation indices cannot be obtained because the original score threshold is not available. The value in bold are the best for each column.

**Table 3:** Performance evaluation results of BVMHC model on non-human species.

	Alleles	Accuracy	AUC	F1	MCC	Specificity	Sensitivity	Precision	AUPR
Class I	H-2-Db	0.829	0.855	0.573	0.466	0.897	0.564	0.583	0.602
	H-2-Dd	0.924	0.870	0.696	0.660	0.975	0.615	0.800	0.751
	H-2-Ld	0.814	0.852	0.698	0.564	0.875	0.682	0.714	0.779
	Mamu-A07	0.905	0.949	0.854	0.783	0.929	0.854	0.854	0.902
	Mamu-A11	0.822	0.899	0.726	0.595	0.880	0.707	0.747	0.805
	Mamu-A2201	0.908	0.957	0.854	0.789	0.955	0.814	0.897	0.943
	Mamu-B01	0.942	0.865	0.667	0.654	0.988	0.550	0.846	0.767
	Mamu-B03	0.857	0.921	0.769	0.666	0.903	0.758	0.781	0.843
	Mamu-B08	0.852	0.911	0.690	0.600	0.875	0.769	0.625	0.776
	Mamu-B17	0.822	0.882	0.717	0.592	0.838	0.782	0.662	0.710
	Mamu-B52	0.827	0.870	0.870	0.617	0.677	0.912	0.832	0.884
	Patr-A0101	0.816	0.838	0.619	0.520	0.935	0.520	0.765	0.688
	Patr-A0401	0.881	0.904	0.636	0.565	0.929	0.636	0.636	0.616
	Patr-A0701	0.825	0.820	0.545	0.438	0.901	0.522	0.571	0.682
	Patr-B0101	0.911	0.947	0.794	0.759	0.991	0.675	0.964	0.894
	Patr-B1301	0.875	0.917	0.903	0.727	0.824	0.903	0.903	0.951
	RT1A	0.893	0.923	0.400	0.352	0.923	0.500	0.333	0.667
Class II	H-2-IAb	0.826	0.797	0.489	0.394	0.925	0.423	0.579	0.627
	H-2-IAd	0.810	0.810	0.571	0.452	0.896	0.533	0.615	0.632

## Data Availability

All data used in this study were obtained from public repositories.

## Supplementary Materials

The following supporting information can be downloaded at: https://www.mdpi.com/article/10.3390/biology11060848/s1, Table S1: Summary of datasets; Table S2: The detailed results based on independent dataset.
